# A simple, less invasive stripper micropipetter-based technique for day 3 embryo biopsy

**DOI:** 10.1186/s40738-016-0027-4

**Published:** 2016-11-25

**Authors:** Luciano Cedillo, Azucena Ocampo-Bárcenas, Israel Maldonado, Francisco J. Valdez-Morales, Felipe Camargo, Esther López-Bayghen

**Affiliations:** 1 Laboratorio de Fertilización In Vitro and Laboratorio de Investigación y Diagnóstico Molecular, Instituto de Infertilidad y Genética, Ingenes México, Carretera México-Toluca No. 5420, Piso 6, Ofna 602 Col. El Yaqui, Del. Cuajimalpa, 05320 Mexico City, Mexico; 2 Facultad de Química, Universidad Nacional Autónoma de México, Ciudad Universitaria, Mexico City, 04510 Mexico; 3 Centro de Investigación y de Estudios Avanzados del Instituto Politécnico Nacional, Departamento de Toxicología, Av. IPN 2508 San Pedro Zac., 07380 Mexico City, Mexico

**Keywords:** Preimplantation genetic screening, Blastomere removal, Embryo biopsy

## Abstract

**Background:**

Preimplantation genetic screening (PGS) is an important procedure for in vitro fertilization (IVF). A key step of PGS, blastomere removal, is abundant with many technical issues. The aim of this study was to compare a more simple procedure based on the Stipper Micropipetter, named S-biopsy, to the conventional aspiration method.

**Methods:**

On Day 3, 368 high-quality embryos (>7 cells on Day3 with <10% fragmentation) were collected from 38 women. For each patient, their embryos were equally separated between the conventional method (*n* = 188) and S-biopsy method (*n* = 180). The conventional method was performed using a standardized protocol. For the S-biopsy method, a laser was used to remove a significantly smaller portion of the zona pellucida. Afterwards, the complete embryo was aspirated with a Stripper Micropipetter, forcing the removal of the blastomere. Selected blastomeres went to PGS using CGH microarrays. Embryo integrity and blastocyst formation were assessed on Day 5. Differences between groups were assessed by either the Mann-Whitney test or Fisher Exact test.

**Results:**

Both methods resulted in the removal of only one blastomere. The S-biopsy and the conventional method did not differ in terms of affecting embryo integrity (95.0% vs. 95.7%) or blastocyst formation (72.7% vs. 70.7%). PGS analysis indicated that aneuploidy rate were similar between the two methods (63.1% vs. 65.2%). However, the time required to perform the S-biopsy method (179.2 ± 17.5 s) was significantly shorter (5-fold) than the conventional method.

**Conclusion:**

The S-biopsy method is comparable to the conventional method that is used to remove a blastomere for PGS, but requires less time. Furthermore, due to the simplicity of the S-biopsy technique, this method is more ideal for IVF laboratories.

**Electronic supplementary material:**

The online version of this article (doi:10.1186/s40738-016-0027-4) contains supplementary material, which is available to authorized users.

## Background

Embryos from women with advanced maternal age that are positive for aneuploidy or genetic disorders, have lower implantation rates and are less likely to achieve live births [1, 2]. Therefore, testing women that are undergoing in vitro fertilization (IVF) for aneuploidies or for genetic disorders can improve Assisted Reproductive Technology outcomes. By selecting chromosomally integral embryos for transfer, patients with advanced maternal age can improve their chances for implantation, reduce pregnancy loss, and considerably reduce the risk of delivering a child with genetic abnormalities [3–8]. There is currently enough evidence provided by a series of randomized controlled trials to support preimplantation genetic screening (PGS) as a reliable tool to improve clinical outcomes in IVF [9–11].

Even though PGS is a sufficient technique to determine aneuploidies and genetic defects, the technique is not without technical issues. A key step in PGS, where many potential pitfalls can occur, is the removal of blastomeres or trophectoderm cells. Although many IVF groups are shifting to trophectoderm biopsy for PGS, cleavage stage biopsy is still a frequently used technique [12–14]. The traditional aspiration technique used for Day 3 biopsy is particularly hard to master, as it requires extensive micromanipulation experience and has been criticized for hindering the capacity of the embryos to reach the blastocyst stage. Buffers used to weaken blastomere-blastomere interactions can diminish embryo integrity with prolonged exposures [15]. Moreover, the time the embryo remains outside the incubator is associated with decreased viability [16].

One of the first steps in performing an embryo biopsy is the creation of a small opening in the zona pellucida. Various methods have been employed, such as partial zona dissection, Acidic Tyrode’s solution, or the laser assisted hatching system [17–20]. Each method has its own risks and benefits. For example, the use of a high-energy laser to remove the zona pellucida, which drastically reduces procedural time, can lead to the destruction of that blastomere or loss of embryo integrity [21, 22].

The conventional aspiration technique used for blastomere biopsy requires the zona pellucida opening to be wide to obtain one blastomere. However, this method is very difficult to master and may cause rupture of the biopsied blastomere and damage to the embryo if not done correctly. The removal of multiple blastomeres can occur, decreasing embryo integrity, or causing complete destruction of the embryo. Other methods have also been suggested, such as displacing the blastomere though the zona pellucida with culture media [19, 23, 24] or pushing against the zona pellucida to squeeze the blastomere out [12–14]. Although these biopsy techniques show similar embryo survival, the displacement method has shown higher survival of the biopsied blastomere [19]. This displacement method has been used in conjunction with partial zona dissection [19] and Acidic Tyrode’s solution [23].

Here, we report that the Stripper micropipette-based biopsy (S-biopsy) technique, when used in cleavage stage biopsy, successfully removed the desired blastomeres for PGS, while considerably reducing the invasiveness to the embryos as well as the performance time in which embryos are manipulated outside their incubators. The main characteristic of this method is the minimal removal of the zona pellucida and the pipetting of the complete embryo. This leads to a pressure imbalance that ejects one blastomere through the small hole in the zona pellucida. This method does not use any additional specialized buffers and can be performed in less time when compared to the conventional method. Therefore, our objective was to characterize and compare the S-biopsy method to the conventional method. We assessed embryo integrity, blastocyst formation, embryo aneuploidy, and procedural time as comparative endpoints.

## Methods

### Study patients and ethical approval

Women were selected for a prospective study conducted at the Ingenes Institute in Mexico City, Mexico between May 2013 and May 2014. The protocol was approved by the Ethics Committee of the Ingenes Institute. Written informed consent was obtained from all patients. Patients were clinically evaluated according to a standardized protocol including personal and family history. The study population consisted of patients who choose to undergo PGS testing for aneuploidy detection (inclusion criteria). A non-complete file or the absence of a signed consent were the only two exclusion criteria. For each patient, the embryos were equally and randomly assigned to either to the S-biopsy method group or the conventional biopsy group.

### Follicle stimulation

All patients were subjected to controlled ovarian stimulation with Gonadotropin-Releasing Hormone agonists and antagonists. The controlled ovarian stimulation protocol consisted in administering a daily dose of a GnRH antagonist (0.25 mg/day; Cetrorelix, Cetrotide, Laboratorio Merck or 0.25 mg/day Ganirelix acetate, Orgalutran Laboratorio Msd) in the luteal phase after menses. Gonadotropins were administered in variable doses (with a minimal daily those of 300 IU), depending on patient age and/or ovarian responsiveness, and further adjusted according to serum estradiol (E_2_) levels and vaginal ultrasound measurements of follicular diameter obtained every 2 or 3 days. Stimulation was prolonged until the mean diameter of leading follicles was >18 mm. Recombinant human Chorionic Gonadotropin (hCG) (Choragon 1000 IU, Laboratorio Ferring) was administered and oocyte retrieval was conducted 36 h after administration of hCG with ultrasound guidance. All 14–18 mm follicles were aspirated (typically 6 to 18), 6 to 14 oocytes were obtained with an average of 10.5 ± 2.5 oocytes per patient.

### Embryo culture

The partner's sperm of the female patient was prepared by density gradient centrifugation, and ICSI fertilization was performed as previously described [25]. The oocytes were all inseminated by ICSI at the same time after hCG administration (around 36 h), and fertilization was judged by the formation of two pronuclei, nineteen hours after insemination. Embryos were cultured in Global Total for Fertilization media (LifeGlobal) and incubated at 37 °C in 8% CO_2_, 5% O_2_, and 89% N_2_. Embryos were evaluated every 24 h and were assessed for development and quality. An Embryologist monitored and recorded all information about fertilization (rate = 67%), embryo development, embryo morphology, transfer and pregnancy for each cohort. Embryo morphology was assessed on Days 2 and 3 by considering the number of blastomeres, symmetry and granularity of blastomeres, type and percentage of fragmentation, presence of multinucleated blastomeres, and degree of compaction. After the biopsy, the embryo culture was extended to Day 5 (Global Total for Fertilization media, LifeGlobal) until Embryo transfer (ET), which was always performed with the optimal embryos.

### Blastomere removal

For both methods, only high quality embryos (<10% fragmentation, symmetrical, and 7–8 cells on Day 3) were assessed on Day 3 (>66 h post insemination). Embryos with multinucleated blastomere may be biopsied if the other morphological parameters were determined to be ideal. All embryos were incubated until Day 5 to assess for embryo integrity and formation of the blastocyst after blastomere biopsy.

For the Conventional Method, selected blastomeres were removed by the standardized protocol. Briefly, the embryos’ cell-to-cell adhesions were weakened by 5 min incubation in a Ca^2+^/Mg^2+^-free bicarbonate buffered G-PGD medium (Vitrolife). Afterwards, the embryos were placed on a holding pipette (K-HPIP-1035, Cook Medical, Bloomington, IN). Using a Hamilton Thorne ZILOS-tk laser (1460 nm, 300 mW), an opening was created in the zona pellucida to expose a complete blastomere, using a 500 μs pulse (at least two parallel laser lines). The single blastomere was aspirated through the hole using an aspiration pipette (MBB-FP-SM-35, Origio, Malov, Denmark). The isolated blastomeres were washed with PBS and placed into a 0.2 mL Fast-reaction PCR tubes (Life Technologies) and kept at −20 °C until analysis. The time required to remove a single blastomere was measured from the attachment of the embryo to the holding pipette to the storing of the blastomere into the PCR tubes.

**Fig. 1:**
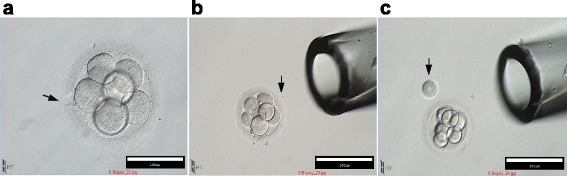
S-biopsy method. With the S-biopsy method, a minimal amount of the zona pellucida is removed (1460 nm, 300 mW for 500 μs) while in the conventional method, a majority of the zona pellucida is removed to expose the blastomere, so that the aspiration pipette can achieve maximal coverage (**a**). The shape of the opening is funneled, minimizing zona pellucida blastomere retention (**b**). The removed zona pellucida region is significantly shorter for the S-Biopsy Method than the conventional method. An appropriate size Stripper-Micropipetter is used to the complete embryo (**b**), leading to the release of the blastomere from the embryo (**c**)

For the S-biopsy method, embryos were placed in Global Total for Fertilization media (LifeGlobal). The laser was positioned at the region of the zona pellucida closer to the selected blastomere. Using a Hamilton Thorne laser, the zona pellucida was breached with three to four 300 μs exposures, leaving a sufficient hole. One or two more laser exposures were performed on the side of the hole to make a funnel-like formation in the proximity of the selected blastomere (Fig. 1). In a laminar flow hood, embryos, while being kept in the same medium droplets, were aspirated and released using a Stripper Micropipetter with a 140 μm capillary (Origio), until spontaneous release of the blastomere was achieved (two or three times). After the blastomere was isolated from the embryo, it was washed using PBS. Each blastomere was transferred to Fast-reaction PCR tubes (Life Technologies) and kept at −20 °C until analysis. For the S-biopsy method, since there is no initial step of attaching the embryo to the holding pipette, the procedural time was measured from locating the embryo to storing the blastomere into the PCR tubes.

### Preimplantation genetic screening

Each blastomere sample was amplified using the SurePlex amplification system (BlueGnome San Diego, CA, USA) according to the manufacturer’s instructions. Comparative Genomic Hybridization was carried out using the 24Sure V3 microarray (Illumina, San Diego, CA, USA) using the protocol described by Fragouli et al. [26, 27]. Amplified DNA was fluorescently labeled (Fluorescence Labelling System, BlueGnome). The samples were co-precipitated, denatured, and analyzed by array hybridization. The hybridization time was 16 h. A laser scanner (InnoScan 710, Innopsys, Carbonne, France) was used to excite the fluorophores and read the hybridization images. The hybridization images were stored in TIFF format and analyzed by the BlueFuse Multi-analysis software (BlueGnome) using criteria and algorithms recommended by the manufacturer. It was possible with this approach to determine the chromosome constitution of each embryo that were categorized as being normal (euploidy) or abnormal (aneuploidy).

### Statistical analysis

The data was determined to be not normally distributed by the D'Agostino-Pearson normality test. Differences between groups were assessed by either the Mann-Whitney test or Fisher Exact test. *P*-values <0.05 were considered significant. All analyses were carried out with use of Sigma Plot software (v. 12.0, San Jose, CA, USA).

## Results

Thirty-eight women met the inclusion criteria and were recruited for this study (average age: 36.4 ± 5.7). After excluding unfertilized oocytes and after Day 3 morphological assessment, 368 high quality-embryos were included. For each patient, all selected embryos were randomly distributed between the two groups. One hundred eighty-eight embryos underwent conventional blastomere removal and 180 embryos underwent the S-biopsy blastomere removal procedure. With the S-biopsy procedure, aspiration of the embryo with a capillary pipette after the minimal destruction of zona pellucida, resulted in release of the selected blastomere (Fig. 1 and see Additional file 1 for a video demonstration).



**Additional file 1** (MP4 20709 kb)


**Fig. 2:**
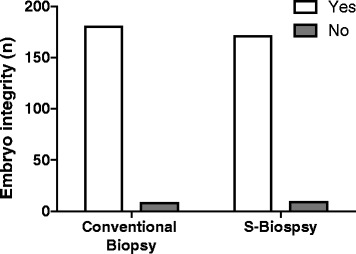
Effects of the biopsy methods on embryo integrity. Hundred-eighty of the 188 embryos biopsied by the conventional method produced single blastomeres while maintaining embryo integrity, whereas the S-biopsy method produced 171 of the 180 embryos. Comparison between the two methods yielded no statistical differences

**Fig. 3:**
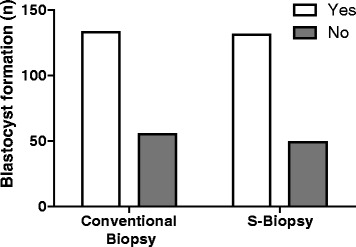
Effects of the biopsy methods on blastocyst formation. From the original 188 embryos biopsied by the conventional method, 133 developed a blastocoel, inner cell mass, and trophoblast tissue. For the S-biopsy method, 131 of the original 180 embryos developed to the blastocyst stage. No statistical differences between the two methods were determined

**Fig. 4:**
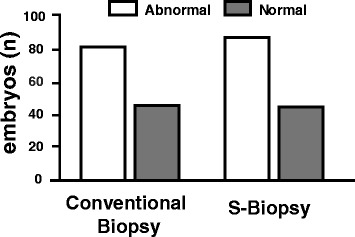
Effects of biopsy methods in aneuploidy results. PGS was performed in a randomly selected subset of embryos: conventional method (*n* = 130) and S-biopsy method (*n* = 135). The amount of abnormal embryos was 82 for the conventional method and 88 for S-biopsy. Four biopsies did not produce any DNA amplification (two in each method). Comparison between the two methods yielded no statistical differences

**Fig. 5:**
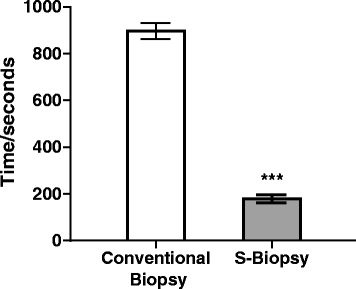
Procedural times for the conventional biopsy and the S-biopsy methods. The time required for the conventional method, as measured from fixing the embryo to the holding pipette to storing the blastomere in a PCR tube, was 897.7 ± 34.0 s. The time required of the S-biopsy method, as measured from the time when the embryo was located to the storing of the blastomere into a PCR tube, was 179.2 ± 17.5 s. Comparison between the methods indicates the time to perform the S-biopsy method was significantly quicker (***, *p* < 0.001, Student’s *t* test)

When comparing the endpoints between the two methods, the S-biopsy method did not result in an increase loss of embryo integrity (Conventional: 95.7%, 95%CI: 91.7–98.0 vs. S-biopsy: 95.0%, 95%CI: 90.6–97.5, Fig. 2). Furthermore, the rate of blastocyst formation, as measured by development until Day 5, was similar between the two groups (Conventional: 70.7%, 95%CI: 63.9–76.8 vs. S-biopsy: 72.7% 95%CI: 65.3–78.3, Fig. 3). A randomly selected subset of embryos for the conventional method (*n* = 130) and S-biopsy method (*n* = 135) were further analyzed for aneuploidies. PGS analysis indicated that aneuploidy rate were similar between the two methods (Conventional: 63.1%, 95%CI: 54.5–70.9 vs. S-biopsy: 65.2% 95%CI: 56.8–72.7, Fig. 4). Overall suggesting, that the embryos are minimally affected by the procedural differences by the two methods. However, the time requirement for each method was significantly different (*p* < 0.001). The average time required for the conventional method was 897.7 ± 34.0 s; whereas, the average time required for the S-biopsy method was 179.2 ± 17.5 s (Fig. 5), leading to a 5-fold decrease in the time the embryo was outside the incubator.

## Discussion

Women of advanced reproductive age have augmented rates of aneuploidy, genetic defects, and miscarriages [28, 29], all of which can be detected with Preimplantation Genetic Testing [30]. Alone, these factors limit 30% of all natural fertilization cycles to reach clinical pregnancy and even less for IVF cycles [31, 32]. Therefore, implementing PGS becomes important to detect aneuploidies that will identify suboptimal embryos. It is worthy to note that PGS only improves the selection of high quality embryos and not the uterine receptivity. Nevertheless, in women fitting the conditions where PGS is considered beneficial, procedure is recommended.

Regardless of the initial debate on the effectiveness of PGS, recent randomized clinical trials have persuasively demonstrated the benefits of PGS in improving embryo selection and implantation rates [9–11]. However, PGS is marred with many technical issues, which makes the optimization of any procedural steps a pragmatic goal [15]. A key caveat for PGS is the need to prevent the reduction in embryo potential associated with the invasive procedures for the removal of a single blastomere [33, 34]. Here, we report a new method that, when compared to the standard conventional method of blastomere removal, requires no additional specialized buffers and decreases the time the embryo is outside the incubator—technical factors associated with decreased embryo quality in IVF.

Environmental stress is a key cause of failures in Assisted Reproductive Technology treatments. Many reports have shown buffers and ion concentrations can affect IVF outcomes [35, 36], suggesting minimizing these effects will improve IVF outcomes. Furthermore, switching between buffers is not preferred. PGS normally uses ion-free buffers to weaken the blastomere intrajunctions; however, it is speculated that good morphological embryos could be damaged, leading to failed pregnancies [37]. Here, one of the benefits of the S-biopsy method was that no specialized buffers were used to weaken the embryo complex; as well, the buffer remained constant. Interestingly, there was no difference in blastomere integrity between the two methods, suggesting the embryo had remained relatively intact.

During embryonic development on Day 3, the embryo will consist of 5 to 10 cells. Due to the undifferentiated nature of these cells, which will give rise to the blastocyst and fetal tissue, this is the ideal time for PGT biopsy. For Day 3 embryonic biopsy, under conventional conditions, the technician normally removes one or two blastomeres, which is sufficient to perform genetic analysis. However, errors sometimes can occur, which leads to the departure of more than two cells of the embryo or the weakening of the embryo complex, affecting its viability [15]. We speculated if a modified stripping procedure would limit this factor. Indeed, under the proposed method, a single blastomere was consistently removed. Furthermore, there was no difference in blastocyst formation between the two methods, which does suggest that the S-biopsy method is equally sufficient as the conventional method at minimally hindering normal embryo development.

Lastly, one of the great advantages of the S-biopsy technique is its short runtime. The time required per an embryo (about 3 min) during the S-biopsy method is a fraction of the conventional method. As pointed out by Zhang et al., decreased time outside of the incubator increases the pregnancy outcomes [16]. We demonstrated a 5-fold decrease in time with a trained technician between the two methods. However, if one considers that usually more than one embryo is prepared at a time, the time required for 5–10 embryos could range between 15 and 30 min for the S-biopsy method and 1–2 h for the conventional method—this estimate does not include manipulation and incubation times, which can significantly lengthen the overall time. Since, it is very important to minimize time during the blastomere biopsy, this does posit the benefit of the S-biopsy method at improving embryo development and pregnancy outcomes.

Our study contains at least three limitations. First, we cannot determine if the S-biopsy method affects implantation rates or clinical pregnancy. Second, one technician preformed all biopsies. It is possible the time required for each method will vary depending on the technician; however, due to the minimized procedural steps and incubation times, the S-biopsy method should always take less time than the conventional method. Third, the S-biopsy method was only performed on high-quality embryos and has not been assessed in embryos of lower quality.

## Conclusion

We propose a method that is equivalent to the conventional method used in PGS for removal of a single blastomere, but uses less time to perform. This will lead to decreased time outside the incubator, benefiting embryo quality, and may lead to improved IVF results.
